# A case report on air bag induced skin burn in a road traffic injury: An experience

**DOI:** 10.1016/j.ijscr.2021.106591

**Published:** 2021-11-15

**Authors:** Nuwadatta Subedi, Suman Baral, Sabita Paudel, Shasi Poudel

**Affiliations:** a Department of Forensic Medicine, Gandaki Medical College, Pokhara, Nepal; b Department of Surgery, Dirghayu Pokhara Hospital, Pokhara, Nepal; c Department of Pharmacology, Gandaki Medical College, Pokhara, Nepal; d Department of Emergency, Fewacity Hospital, Pokhara, Nepal

**Keywords:** Air bag deployment, Burn, Case report

## Abstract

**Introduction:**

Air bag deployment after high velocity trauma has been associated with burn injuries. So, we aimed to present a clinical case report associated with air bag deployment experienced by the author himself.

**Case presentation:**

The author was driving a hatchback car which collided head on with the high speeding vehicle from opposite direction. He sustained a burn injury around 4 × 3 cm in size in the flexor aspect of right forearm involving epidermis and some part of dermis which was superficial partial thickness in nature when the air bag deployment was observed at both the sides. Burn injury was healed with topical antibiotics and regular dressings with no any complications.

**Discussion:**

Air bag deployment has always been a safety measures for the road traffic injuries but the safety comes with a cost. It has been associated with burn injuries, especially chemical induced, thermal and frictional burns. Timely diagnosis of type of burn and intervention is required in order to minimize complications associated with burns.

**Conclusion:**

Though burn injuries associated with air bag deployment cause less harm or complications, the companies making such commodities should explore the further options in order to develop burn injury free vehicle safety.

## Highlights

• Air bag introduction has been one of the milestones for road traffic safety.
• Air bag deployment is associated with thermal, chemical and frictional burn injury.
• Identification of type of burn is required for proper management.

## 1 Introduction

An air bag is a vehicle occupant-restraint system using a bag designed to inflate extremely quickly, then deflate immediately during a collision. Designed to reduce fatalities in high velocity trauma, burns related to these bags are seen and commonly affect arms, hands, face and eyes [1]. Most burn injuries are minor though some of them may be life threatening leading to spinal, aortic and chest injuries [2]. Thermal, chemical and frictional burns have been attributed to air bag deployment injury and most of them are found to be superficial to superficial partial thickness burns. It has been estimated that burns account for nearly 8% of injuries that occur due to air bag deployment [3]. As of 2015, National Highway Traffic Safety Administration estimates that 44,869 lives have been salvaged due to frontal air bags [1].

We present a case of high velocity road traffic injury where the author sustained a superficial burn on the flexor surface of right forearm. We believe this to be the first ever reported case of air bag deployment burn injury from this region. The work has been reported in line with the SCARE 2020 criteria [4].

## 2 Case report

**Fig. 1:**
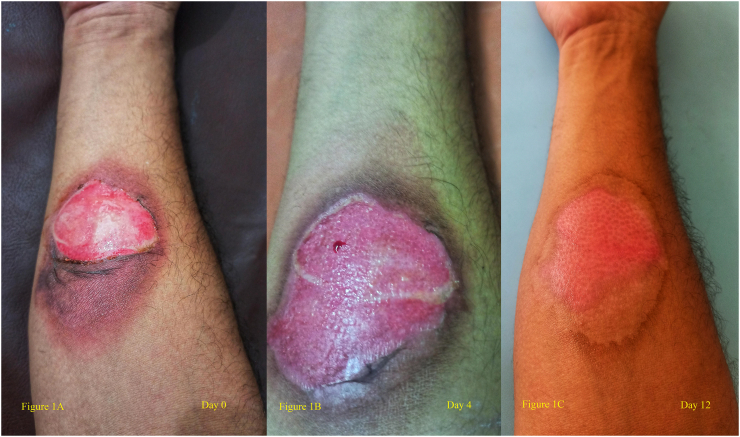
**Figure 1A** shows the burn injury size at Day 0 measuring 4 × 3 cm involving the flexor aspect of right forearm with involvement of epidermis and some part of dermis. **Figure 1B** shows the development of granulation tissue and healing of the wound at Day 4. **Figure 1C** shows the healed wound at Day 12.

**Fig. 2:**
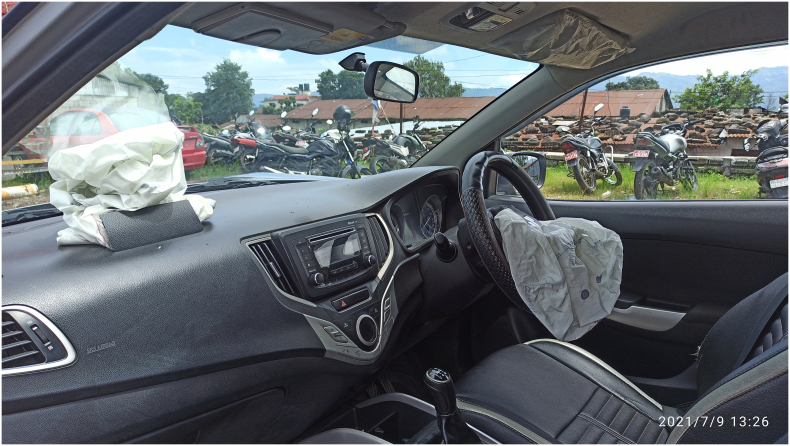
Figure 2 shows the deployment of air bags at both the sides (Driver side and passenger side).

The author (NS) was driving in his hatchback car along with passenger on the other side at the speed of 20 km/h when he sustained a head on collision with an over speeding van from opposite direction. Air bags were deployed from the front on both the seats (Fig. 2). The author (NS) sustained burn injury at the flexor aspect of right forearm (Fig. 1A) whilst the other author (SP), the front seat occupant had sustained contusions on the lower abdomen as seat belt marks. The other vehicle had wrecked at the front part without any significant injury to the driver. Emergency assessment at the nearest hospital casualty showed clinically stable vitals, spasms along the cervical region with no evidence of major injuries to any involved in the accident. The author's burn injury due to deployed air bag was about 1% involving epidermis and some part of dermis (second degree burn). The size of the wound was 4 × 3 cm along with scraping of the epidermal layer which was addressed with dressing, application of antibiotics ointment (Neosporin), and oral antibiotics for 5 days. The burn injury gradually healed without any evidence of infection (Fig. 1B, C).

## 3 Discussion

Air bag deployment during high velocity trauma has always been considered an important step for prevention of major fatal injuries-whilst this technology never comes without any cost since their introduction in early 1970s [1]. The role of protection by an airbag is already proven, but at times, it is associated with the adverse effects of its inflation which is underestimated [5]. We have reported this case to add to the existing literature so that the design of the airbags can be further refined to minimize the harms associated with its mechanism. This can also imply to opt for necessary precautions to minimize the injuries due to the existing mechanisms of airbag inflation.

Air bag related injuries do occur in the form of skin abrasions to spinal fractures, thoracic injuries or aortic dissection which sometimes proves to be fatal, though potential benefit outweighs the harm during traumatic injuries [1], [2]. Within 10 m seconds of the impact, air bags get deployed leading into the exothermic chemical reaction within the bag which inflates it and creates a cushioning effect reducing the force of impact to vehicle structures [6]. This also absorbs the energy, and decelerates the occupant in the vehicle [7]. Around 8% of the injuries are related to air bag deployment burns which occurs through mechanism like thermal effects due to high temperature gases, chemical burns due to particulate materials and alkaline corrosives which includes sodium hydroxide, and burns related to frictional injury [3], [8].

Composed of rubber lined nylon, air bags get deployed in various phases. Detection phase, which when deceleration occurs, activates the cartridge of sodium azide which is a highly reactive chemical which leads to chemical burns when comes in contact with bodily fluids like sweats and tears. Second phase, termed as inflation occurs within 100 ms where combustion of sodium azide releases alkaline byproducts along with nitrogen gas, carbon-dioxide, metallic oxides and sodium hydroxide which act as extreme corrosive and may lead to chemical burns when it comes in contact to skin. Third phase includes deflation that occurs within 2 s when the gaseous filled bag gets deflated and vents the hot gaseous air with maximum temperature reaching up to 500 °C depending upon the types of air bag which might cause thermal burn when contacting the skin [5], [9].

Anatomically, the most common site of burn remains the upper extremities which had been seen in almost 42% of the studies reported which is similar to our case too [1]. Also, the depth of involvement remained the superficial partial thickness burn which have been mentioned in other studies too [1], [3], [10]. Thermal burn occurs via two mechanisms- indirect injury occurs when the clothing melts and burn down the skin whilst direct injury is evident due to direct effect of heat produced in the skin. Hands, arms and face were common sites for thermal burn as proposed by Hallock et al. where he suggested could be due to the upper extremities being pushed forward and away from the air bag, causing the forearms and hands to particularly be in harm's way of the hot air [8]. Treatment includes the antibiotics ointment use, regular dressing as most of the thermal burns heal without scarring or minimal complications that highlights the good prognosis [1].

Chemical burn due to alkaline aerosols occur less frequently in comparison to thermal burns but the potential for deep tissue burns exceed. Superficial burns are seen as painful areas of red-purplish erythema whilst deeper chemical burns appear as well-demarcated areas with a distinct splash shape [5]. The alkaline chemicals released cause damage of cell membrane through saponification of fatty acids and derangement of mucopolysaccharide, protein degradation and increased blood flow at burn site acts as an ideal medium for bacterial growth [11]. Importance of pH measurement has been mentioned in literatures which illustrates the pH of the burnt site to be more than 7 [12]. Diagnosis of chemical burn has always been vital as accurate diagnosis and prompt treatment plays a critical role in order to minimize the complications. Treatment of chemical burn requires immediate irrigation of the burn with saline, if possible, at the site of trauma as delay might cause deeper involvement of the tissues. Application of silver sulphadiazine, antibiotics and even corticosteroids have been mentioned in literatures. These lesions heal without major complications provided that rapid treatment is available [12].

Various measures play a role to minimize the air bag induced burns. Avoiding metal accessories in steering wheel, use of larger vents which is positioned away from commonly burned areas like upper extremities and arms that help to minimize the heat flux per unit area, newer advancements to look for fewer inflammable chemicals to be used in the bags and avoidance of synthetic clothing, gloves whilst driving in high velocities could be some of the measures that could be enacted to ensure safe ride [13]. The preventive strategies could be a safe riding, no over speeding in the high ways, and switching off the devices when riding at lower velocities especially at city areas with heavy traffic [7], [14].

## 4 Conclusion

Air bags deployment are associated with burn injuries. So, it is vital to recognize the type of burn and manage accordingly to minimize the complications associated with it.

## Provenance and peer review

Not commissioned externally peer reviewed.

## Funding

This case report did not receive any specific grant from funding agencies in the public, commercial, or not-for-profit sectors.

## Ethical approval

Ethical approval was not mandatory for publication of case reports as per the institutional policy.

## Consent

“Written informed consent was obtained from the patient for publication of this case report and accompanying images. A copy of the written consent is available for review by the Editor-in-Chief of this journal on request”.

## Guarantor

Suman Baral

## CRediT authorship contribution statement

Design and Idea: Nuwadatta Subedi, Suman Baral, Shasi Poudel

Drafting: Suman Baral, Nuwadatta Subedi, Sabita Paudel

Final Revision: Suman Baral, Nuwadatta Subedi, Shasi Poudel, Sabita Paudel

## Declaration of competing interest

Authors declare that there are no any conflicts of interest regarding publication of the manuscript.
